# Self-organized iron-oxide cementation geometry as an indicator of paleo-flows

**DOI:** 10.1038/srep10792

**Published:** 2015-06-30

**Authors:** Yifeng Wang, Marjorie A. Chan, Enrique Merino

**Affiliations:** 1 Sandia National Laboratories, P. O. Box 5800, Albuquerque, New Mexico 87185-0779, USA; 2 Department of Geology and Geophysics, University Utah, Salt Lake City, Utah 84112-0111, USA; 3 Department of Geological Sciences, Indiana University, Bloomington, Indiana 47405, USA

## Abstract

Widespread iron oxide precipitation from groundwater in fine-grained red beds displays various patterns, including nodulation, banding and scallops and fingers. Hematite nodules have been reported also from the Meridiani Planum site on Mars and interpreted as evidence for the ancient presence of water on the red planet. Here we show that such patterns can autonomously emerge from a previously unrecognized Ostwald ripening mechanism and they capture rich information regarding ancient chemical and hydrologic environments. A linear instability analysis of the reaction-transport equations suggests that a pattern transition from nodules to bands may result from a symmetry breaking of mineral dissolution and precipitation triggered by groundwater advection. Round nodules tend to develop under nearly stagnant hydrologic conditions, while repetitive bands form in the presence of persistent water flows. Since water circulation is a prerequisite for a sustainable subsurface life, a Martian site with iron oxide precipitation bands, if one were found, may offer a better chance for detecting extraterrestrial biosignatures on Mars than would sites with nodules.

Fine-grained sandstone in Mesozoic red beds of the Colorado Plateau in the southwestern United States contains iron oxide cements (mainly hematite and goethite). Some are early diagenetic grain coats and films that impart an overall red-bed appearance and others are late diagenetic features that display spectacular repetitive patterns, including evenly spaced nodules and repetitive bands with nested scales spanning two to three orders of magnitude (Fig. 1; Supplementary Figure S1). These nodules are commonly referred to as concretions, which are cemented mineral masses. The size of concretions typically ranges from millimeters to centimeters (Fig. 1A), while the spacing of bands ranges from millimeters to sub-meters (Fig. 1B). Spatial transition of one pattern to another (Fig. 1C) or one pattern superimposed on another (Fig. 1A) is also observed (Fig. 1A, C, D). These patterns may embed important information about paleo-diagenetic environments, especially regarding paleo-fluid migration and reactions^1^,^2^. Field evidence indicates that the formation of iron oxide precipitation bands in sandstone may be closely related to groundwater flows^1^,^2^. Concretions formed in Jurassic Navajo Sandstone have been proposed as a terrestrial analogue to hematite spherules detected by the rover Opportunity at the Meridiani Planum site on Mars^3^,^4^.

Repetitive iron oxide precipitation bands in rocks have generally been lumped as “Liesegang bands” without further elaboration of underlying mechanisms. Typical Liesegang bands are thought to form by counter-diffusion of two reactants via a nucleation-growth-depletion mechanism^5^,^6^ or post-nucleation Ostwald ripening^7^,^8^. These theories, however, are unable to account for key features of iron oxide precipitation bands observed in sandstone. First, it seems that the nucleation-growth-depletion mechanism is unlikely, simply because iron oxide precipitation in red-bed sandstone is usually pervasive, suggesting that nucleation was not a limiting factor for the initiation of individual bands (Fig. 1B, C). Furthermore, the spacing between two neighboring bands in a specific packet of bands is observed to be more or less constant (Fig. 1B), contrary to the exponentially increasing banding spacing predicted from the counter-diffusion Liesegang banding theory^9^. Also, the spacing of the bands in some cases can reach sub-meters, which apparently exceed a typical scale of Liesegang bandings that can be produced in a diffusion-dominated system. In addition, it is hard to imagine that a counter diffusion process alone would be able to generate iron oxide bands over significant volumes of sandstone (up to tens of meters vertically and kilometers laterally). We postulate that these large volumes of banded sandstone must have involved regional groundwater flows, as suggested by field observations^1^,^2^. Other mechanisms have also been proposed to explain the iron oxide patterning. For example, the formation of iron oxide nodules has been attributed to microbial oxidation, which could be induced when an Fe(II)-containing fluid is mixed with an oxidizing fluid^10^,^11^. But how this reaction becomes periodically localized in space remains unexplained. In addition, the environment for red bed deposition and diagenesis is generally poor in organic matter and microbial activity may be limited^12^. Importantly, no existing theory can explain a geometrical transition from one pattern to another, for example, from nodulation to bandings or vice versa.

The field evidence seems to indicate a self-organizational origin of iron oxide precipitation patterns^13^,^14^, whereby patterns arise from the internal dynamics of the water-rock system, without external periodic forces or preexisting templates. This assertion is supported by the observation that iron oxide nodules or bands cut cross the original eolian laminae (Fig. 1B and Figure S1). In this paper, we show that iron oxide precipitation patterns in fine-grained sandstone can autonomously emerge from a previously unrecognized Ostwald ripening mechanism. We further show that a geometric transition from one pattern to another can be triggered by a change in groundwater flow velocity. Thus, these patterns can potentially capture important information regarding paleo-flows. This work provides a new perspective for interpreting geochemical and hydrological signatures of widespread iron oxide cementation patterns including those detected on Mars.

## Results

### Ostwald ripening in confined pore space

**Figure 1:**
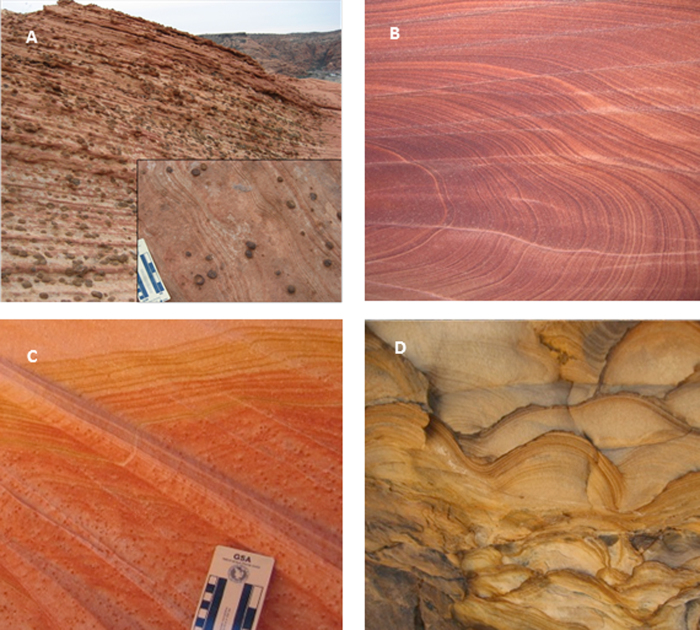
Formation of iron oxide concretion nodules, bands and scallops in Navajo eolian sandstone. **(A**) Iron oxide concretions (several cm in diameter) in cross-bedded host rock at Snow Canyon, southwestern Utah. Nodule formation is superimposed on eolian laminae and has periodic banding (insert). (**B**) Very fine (~mm-thick) diagenetic iron oxide bands cut across the original eolian laminae (approximately horizontal) at Coyote Buttes, northern Arizona. Image width ~20 cm. (**C**) To the right of center note the spatial transition from bands (above) to small concretions at Coyote Buttes, northern Arizona. (**D**) Multiple “scallop” iron oxide bands at Calf Creek, Grand Staircase Escalante National Monument, Utah. Image width ~25 cm.

**Figure 2:**
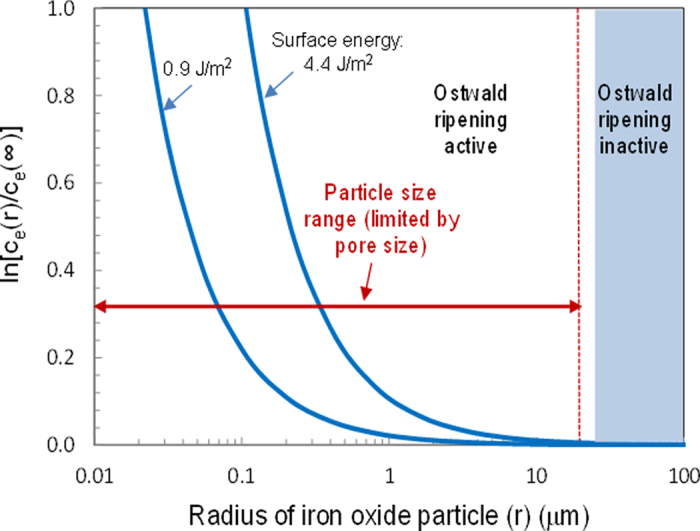
Effect of pore confinement on Ostwald ripening of iron oxide in fine-grained sandstone. Ostwald ripening tends to occur in a particle size range where the solubility of a solid phase highly depends on the size of solid particles. In fine-grained sandstone, the particle size of iron oxide cement is limited by the pore size of the rock, rendering this type of rock to be an ideal system for Ostwald ripening. The boundary between the active and inactive regions is drawn at the particle size (~50 micrometers in diameter) corresponding to the solubility of the particle being ~0.1% of that of the bulk phase. The range of surface energy for iron oxide, 0.9–4.4 J/m^2^, is taken from reference 60. The typical pore size range for Navajo Sandstone is ~40 micrometers in diameter^18^.

Self-organization requires a positive feedback among physical and chemical processes involved in a system^15^. We here propose a new mechanism for the pattern formation of iron oxide precipitation in fine-grained sandstone by considering Ostwald ripening confined in small pore space and coupled with groundwater advection. In Ostwald ripening, larger particles grow even larger at expense of smaller particles because of the size dependence of particle solubility^16^:


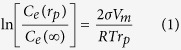


where *C*_*e*_(*r*_*p*_) is the solubility of a particle with a radius *r*_*p*_; *C*_*e*_(∞) is the solubility of the bulk solid phase; σ is the surface energy between iron oxide and water; *V*_*m*_ is the molar volume of the solid; *R* is the gas constant; and *T* is the absolute temperature. Ostwald ripening is effective only for small particles, less than 50 micrometers in size, so that a slight variation in size would result in a significant solubility difference between two nearby particles (Fig. 2). Once the particle size of iron oxides becomes sufficiently large, Ostwald ripening ceases, and a pattern formed would become arrested. However, the size of iron oxide precipitate particles is limited by the sandstone pore size, which is typically smaller than tens of microns^17^,^18^. Note that a typical particle size of iron oxide in Navajo Sandstone is ~10 mcrometers^3^. Thus, iron oxide precipitates confined in these pores remain effective almost forever for Ostwald ripening. Also, because of their high surface energies^19^, according to Equation (1), iron oxides (or hydroxides) are expected to have a higher tendency than other authigenic minerals for Ostwald ripening. All these combined lead to widespread iron oxide pattern formation in eolian sandstone, as observed (Fig. 1)^3^,^4^. The proposed Ostwald ripening mechanism is consistent with iron isotopic measurements from Navajo Sandstone, which indicate that iron oxide nodules formed through simple mineral dissolution and precipitation^20^.

The dependence of iron oxide solubility on particle size in sandstone is more complicated. Experiments suggest that porous host media could significantly affect precipitation pattern formation^21^. Iron oxide precipitates form coating films on sand grain surfaces^22^,^23^. Based on the Ostwald ripening principle, a thicker film [equivalent to larger particles in Equation (1)] has a lower solubility than a thinner film and therefore tends to grow even thicker at expense of the latter. However, this tendency is countered by other factors. In a coating film, a precipitate can interact strongly with their neighbors, depending on the coverage of the coating or the thickness of the film^24^. As new iron oxide crystallites are added, a sharp variation film thickness in the neighborhood would result in additional stress in the film or even create a new interface, thus adding energy to the film. This variation would also change the curvature of iron oxide-water interface, thus modifying the solubility of the solid. Furthermore, due to the pore confinement, the growth of a precipitate tends to push a neighboring particle away to make room for growth, which creates a crystallization force on its neighbors and also modify the solubility of the solid^25^,^26^. All these effects amount to smooth out a spatial discontinuity in film thickness or mineral content distribution. Therefore, it is reasonable to assume that the solubility of iron oxide at a given point 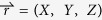 depends not only on the local film thickness but also on its distribution within the neighborhood. To capture these effects, we assume that the excess Gibbs free energy (Δ*G*_s_) of an iron oxide film relative to the bulk phase at a given point is a monotonic decreasing function of the weighted iron oxide content within the neighborhood (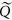): 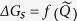 with 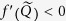. One reasonable choice for the function could be that 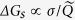, in analogue to Equation (1). The weighted iron oxide content 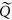 can be related to the local iron oxide content in bulk rock *Q* by:


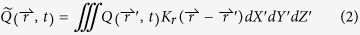


where 
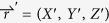
 is the coordinate vector for a point in the neighborhood; *K*_*r*_ is a kernel function with 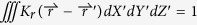, a weighting function that describes the textural dependence of the Gibbs free energy of iron oxide at a location 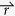 on the iron oxide content distribution in the neighborhood. The kernel function *K*_*r*_ is assumed to be isotropic and independent of location, and it vanishes as 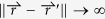 as it is expected that the textural dependence diminishes as the two spatial points become further apart. Based on the Curie principle, by expanding the term *Q* in Equation (2), we can approximate Equation (2) by^27^,^28^:


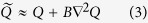


where *B* is a constant characterizing the influence of mineral distribution in the neighborhood of a specific point on mineral solubility at that point. The weighted iron oxide content at a point is expected to increase with the mass in the neighborhood, and *B* must be positive. The solubility of iron oxide (*C*_*e*_) at 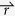 can then be expressed as:


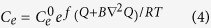


where 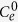 is the solubility of the bulk phase. The precipitation rate (*R*_*p*_) of iron oxide is assumed to follow the first order kinetics:


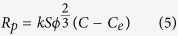


where *C* is the dissolved iron concentration in pore water; *k* is the reaction rate constant; *φ* is the porosity; and the term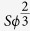 represents the specific pore surface area available for iron oxide precipitation. Term 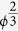 converts the pore volume to the pore surface area with *S* as a geometric factor of the pores. We assume that iron oxide cementation proceeds through coating the pore surfaces^22^,^23^. Iron oxide cementation in sandstone can then be described by:


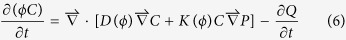



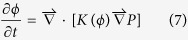



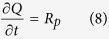



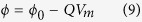


where *P* is the hydraulic head; and *φ*_0_ is the porosity of sandstone without iron oxide cementation. The effective diffusion *coefficient* D(*φ*) and the hydraulic conductivity *K*(*φ*) are described by *D*(*φ*) = *D*_0_*φ*^*m*^ and 
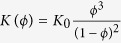
, respectively^29^,^30^,^31^, where *D*_0_ is the diffusion coefficient of dissolved iron species in pore water and both *m* (≈2, ref. 30) and *K*_0_ are constants.

### Pattern formation and symmetry breaking

**Figure 3:**
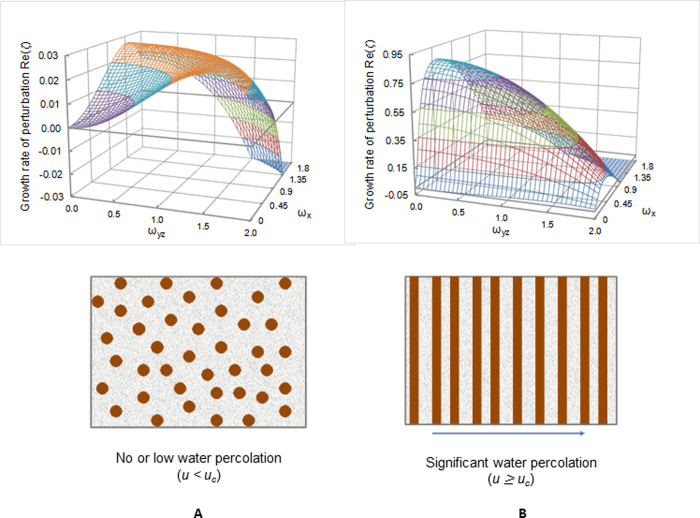
Emergence of iron oxide cementation patterns predicted from a linear stability analysis. The wave number with the maximum growth rate (*ω*_*max*_) determines the geometry and scale of the emerging pattern. Groundwater is assumed to percolate along the x-direction. *ω*_*x*_ is the wave number in the direction parallel to the groundwater flow and *ω*_*yz*_ is the wave number in the direction perpendicular to the flow. In a case with no significant flow present, the growth rate of perturbation has equal maxima on both *ω*_*x*_ and *ω*_*yz*_; therefore a precipitation pattern forms – i.e., spots or nodules – with no directional preference (**A**). But for significant or dominant water flow, the growth rate of perturbation displays a maximum only at *ω*_*yz*_ = 0; consequently a pattern forms only along the flow path, while patterns along the direction normal to the flow direction are suppressed (**B**) since *ω*_*max*,*yz*_ = 0, as illustrated schematically in the lower panel. *α *= 0.013, *φ*_*s*_ = 0.15, *θ* = 1.0, *η* = − 1.0, *β* = 0.2, *m *= 2, and *u* = 0 (left) or 6 (>u_c_ ≈ 1) (right).

We now want to show that an initially homogenous system can self-organize to form a repetitive pattern from infinitesimal fluctuations (or perturbations). We have performed a linear stability analysis of Equations (4), (5), (6), (7), (8) for a sandstone formation initially with a uniform distribution of iron oxide content (*Q*_*0*_) and porosity excluding iron oxide (*φ*_0_). Parameter *φ*_0_ remains constant during pattern formation. In the presence of an advective flow, water is assumed to percolate along x-axis. In the analysis, we first introduce a perturbation of wave number vector (*ω*_*x*_, *ω*_*y*_, *ω*_*z*_) around a uniformly distributed steady state and then determine the growth rate (ζ) of the perturbation as a function of the wave number vector (see Methods):


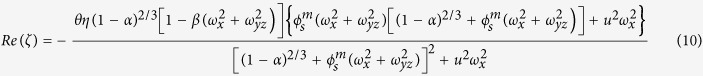



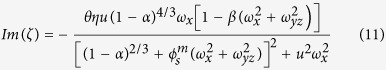


where *θ *= *f*(*Q*_0_)/*RT*; *η = f*′(*Q*_0_)*Q*_0_/*f*(*Q*_0_) < 0; *α *= *Q*_0_*V*_*m*_/*φ*_0_; *β *= *B*/*L*^2^; *φ*_*s*_ = *φ*_0_(1–*α*); *ω*_*yz*_ is the wave number along the direction perpendicular to the water flow 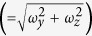; and *u* is a scaled flow velocity (see Methods). The real part of *ζ*, *Re*(*ζ*), represents the growth rate of the perturbation, while the imaginary part, *Im*(*ζ*), characterizes a temporal oscillatory behavior of the perturbation^27^,^32^. A positive *Re*(*ζ*) value indicates that the perturbation with the corresponding wave number tends to grow with time, leading to the emergence of a spatially repetitive pattern^32^. The wave number vector (*ω*_*max,x*_, *ω*_*max,y*_, *ω*_*max,z*_) corresponding to the maximum growth rate determines the periodicity of the pattern actually formed. Note that this preferred wave number vector is independent of parameters θ and η, implying that the actual form of function *f*(*Q*_0_) has no effect on the periodicity of the pattern as long as *f*′(*Q*_0_) < 0. Figure 3 shows the general behavior of *R*e(*ζ*) as a function of the wave vectors.

**Figure 4:**
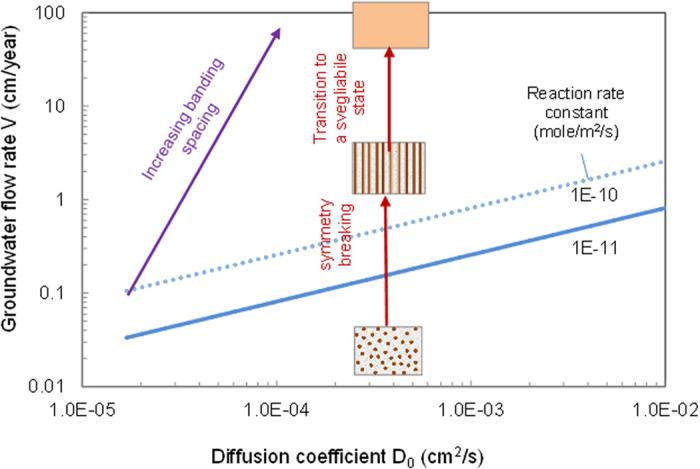
Symmetry breaking and pattern transitions of iron oxide cementation. As the groundwater flow rate increases from zero, the cementation pattern “breaks” in symmetry from nodule formation to banding when crossing the boundary shown for two values of the reaction rate constant, 10^−10^ and 10^−11^ mole/m^2^/s. With further increase of the flow rate, the banding spacing becomes so large that the banding patterns disappear from our observational domain, entering a *svegliabile*^33^ state where iron oxide precipitation becomes relatively uniform. *φ*_*s*_ = 0.15, 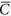 = 5 × 10^−10^ mole/cm^3^ solution, 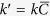 = 10^−11^ - 10^−10^ mole/m^2^/s, *Q*_0_ = 10^−3^ mole/cm^3^ bulk sediment, 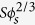 = 0.^2^ m^2^/cm^3^ bulk sediment, and *u*_*c*_ ≈ 1.

The linear stability analysis shows that a spatially repetitive pattern can autonomously emerge from the pore-space-confined Ostwald ripening, as indicated by the existence of positive *R*e(*ζ*) values for certain wave numbers in Fig. 3. In a no or low groundwater flow case, the preferred wave number is identical in all directions, implying the formation of a spatially isotropic pattern (i.e., the formation of spots or nodules; Fig. 3A). Since *Im*(*ζ*) = 0, the pattern formed would be stationary. Whereas, in the presence of significant groundwater advection, *ω*_*max,yz*_ = 0, which implies an infinite wave length in the direction perpendicular to the flow, implying that the periodic iron oxide precipitation is suppressed in that direction and repetitive banding can form only along the flow path. Since *Im*(*ζ*) ≠ 0, the iron oxide content at a given spatial point would oscillate with time. The only way to have this situation is that the pattern formed would slowly move downstream, forming a chemical wave (Fig. 3B). Because of this chemical wave, iron oxides in fine-grained sandstone may be much more mobile than we thought before. As discussed below, with increasing the flow rate, the spacing of the banding increases and eventually become so large (i.e., *ω*_*max,x*_ → 0) that the bands disappear from our observational domain. The system then enters a *svegliabile* (dormant) state^33^, in which iron oxide cementation become relatively homogeneous within the observational domain. Large red and bleached patches observed in some sandstone^34^ may represent a *svegliabile* state^33^. This state can be “awaken” (i.e. become patterned) as the flow rate decreases at a later stage of a water flow regime, leading to an overprint of repetitive patterns on a previously uniformly cemented domain (Fig. 1D). The behaviors in pattern transition are summarized in Fig. 4. Given a typical range of *D*_*0*_ (5 × 10^−5^ – 5 × 10^−4^ cm^2^/s), the critical flow rate for the symmetry-breaking transition from nodules to repetitive bands is estimated to be very low, <0.2 cm/year (Fig. 4), implying that the occurrence of iron oxide nodules may indicate a practically stagnant hydrologic condition, consistent with the field observation at Lake Brown, Western Australia, where hematite nodules precipitated in a shallow sediment and groundwater was static^35^,^36^. At much higher flow velocities, around 10 meters/year, another symmetry-breaking transition takes place (Fig. 4), from banding behavior to a so-called *svegliabile* (or dormant) state noted above (top box in Fig. 4).

### Reconstruction of paleo-flow fields

A sensible model must be able to predict the scale of patterns formed. The wave length of a cementation pattern can be estimated by (see Methods):


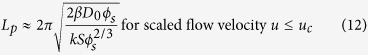



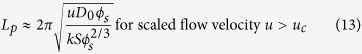


where *u*_*c*_ is the critical scaled flow rate for a transition from nodule formation to banding. Note that parameter β characterizes the influence range of neighboring iron oxide precipitates, estimated to be on the order of a few sediment grains to the size of an individual iron oxide concretion or band (*β *= ∼ 0.1–1.0). For a stagnant flow case, taking the typical parameter values: *φ *= 0.1–0.3 (ref. 23), 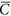 = 10^−10^–10^−9^ mole/cm^3^ solution^23^,^37^, *D*_*0*_ = 5 × 10^−5^ – 5 × 10^−4^ cm^2^/s (accounting for the potential effect of mechanical dispersion)^38^, 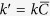 = 10^−11^ – 10^−10^ mole/m^2^/s (ref. 39), 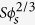 = 0.02–0.2 m^2^/cm^3^ bulk sediment^40^, we estimate the wave length of a pattern to be 0.1 to 30 cm, consistent with field observations (Fig. 1)^3^,^4^,^13^,^23^,^41^. In an advective flow-dominant case, according to Equation (13), the spacing of the banding can vary greatly from millimeters to sub-meters, depending on the actual flow rate involved. Therefore, according to the theory presented here, the geometry of iron oxide cementation patterns provides rich information about paleo-diagenetic environments. The chemical wave concept introduced here provides a logical explanation for the observed positive correlation between the size and the spacing of concretions^3^, because both parameters are supposed to be proportional to the wave length.

Since 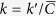 (where *k’* is in unit mole/m^2^/s), from Equations (12) and (13), we have: 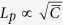. The scale of a pattern thus increases with the dissolved iron concentration. In general, a high dissolved iron concentration would enhance pattern formation, because mass transfer from one location to another is an important step for the proposed Ostwald ripening mechanism. The solubility of bulk iron oxides is known to be extremely low (~nanomolar)^23^. The dissolution of iron oxide precipitates in fine-grained sandstone can be enhanced by several factors, including the small size of the precipitates due to pore confinement (Fig. 2), the presence of Fe^3+^-complexing ligands^37^, and low pH conditions^37^. Sulfate (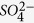) seems to play an important role in Fe^3+^ mobilization, due to its ability to complex with ferric iron^42^, and also because sulfate-rich waters in nature are usually acidic^43^. At Lake Brown in Western Australia, hematite nodules were found to precipitate in shallow subsurface sediments^36^. The size of nodules ranges from 2 mm to 4 cm. The waters involved in nodule formation are Na-Cl-Mg-SO_4_-rich acid saline waters with a 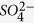 concentration ranging from 1,600 to 3,300 ppm and a pH ranging from 3.5 to 4.3. Similarly, hematite spherules ~10–100 μm in diameter were found to precipitate in sulfate-rich rocks formed by aqueous, acid-sulfate alteration of basaltic tephra on Mauna Kea Volcano, Hawaii^44^. Note that sulfate-rich acidic waters can be formed from chemical divides driven by water evaporation^43^. This may explain why red beds and the associated repetitive iron oxide precipitation patterns seem likely to occur in hot and arid regions^12^.

**Figure 5:**
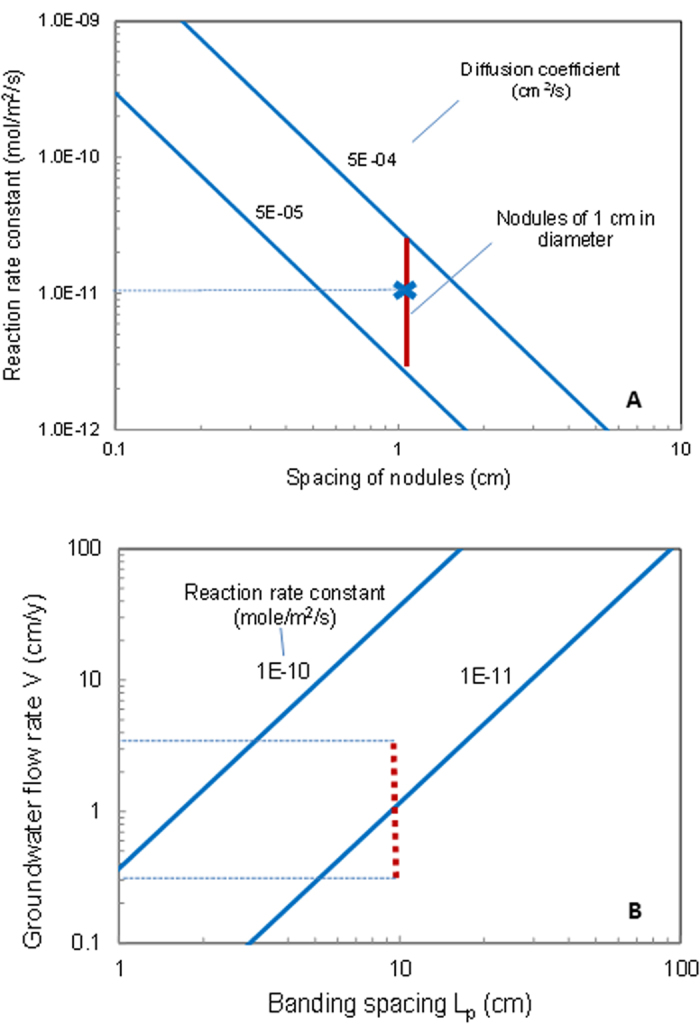
Estimation of key chemical and hydrologic parameters from iron oxide cementation patterns. The rate constant for iron oxide precipitation can be estimated from the spacing of nodules using Equation (12) (**A**). Symbol x is the laboratory measurement of hematite dissolution rate^39^. For a given reaction rate constant, the groundwater flow rate can be estimated from the spacing of iron oxide bands using Equation (13) (**B**). Note that Equation (13) is valid only for relatively large flow rates. Vertical bars indicate the ranges of reaction rate constant and groundwater flow rate estimated from pattern sizes. *φ*_s_ = 0.15, 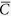 = 5 × 10^−10^ mole/cm^3^ solution, 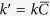 = 10^−11^ mole/m^2^/s, *Q*_0_ = 10^−3^ mole/cm^3^ bulk sediment, 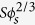 = 0.^2^ m^2^/cm^3^ bulk sediment, *β* = 0.2, and *D*_*0*_ = 510^−5^ cm^2^/s (**B**).

Interestingly, the model developed above may allow us to reconstruct key paleo-environmental parameters from the geometry of cementation patterns. For illustration, let’s focus on two important parameters: chemical reaction rate constant and groundwater flow rate. We can first constrain the reaction rate constant from the size of nodules. If the spacing of nodules is 1 cm, from Equation (12), we estimate the reaction rate constant for hematite dissolution to be ~3 × 10^−12^ – 3 × 10^−11^ mole/m^2^/s (Fig. 5A), comparable with laboratory measurements (~10^−11^ mole/m^2^/s, ref. 39). With the estimated value, Equation (13) can then be used to constrain the groundwater flow rate for the formation of repetitive iron oxide precipitation bands (Fig. 5B). For example, a banding spacing of 10 cm may indicate a flow rate of 0.3 – 2.5 cm/year. The absolute estimated value of flow rate may be subject to large uncertainties, because it depends on the values chosen for a number of other parameters, especially for a typical dissolved iron concentration (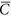), which could vary significantly as discussed above. Nevertheless, the concept developed here can allow us to reconstruct relative flow rates fairly accurately. This is because, for a given sandstone formation, all parameters, except scaled flow rate (*u*), in the right hand side of Equation (13) are expected to remain relatively constant. The relative flow rate is then directly proportional to 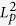. Note that cementation bands form normal to the flow direction (see the discussion above). A careful mapping of cementation patterns and banding spacing over rock outcrops can potentially allow us to reconstruct a past water flow field for a sandstone aquifer.

The model presented here predicts other salient features of iron oxide cementation patterns in sandstone as well. The iron oxide cementation patterns studied here occur in two symmetries, bands and nodules, that are able to grade into each other in the field (Fig. 1C), and that are – according to our model – linked genetically to the velocity of groundwater flow, as shown in Figs. 3 and 4. The ability of the linear instability analysis to predict the two symmetries is one of the strengths of the model. From the model, it is expected that the banding spacing resulting from the proposed mechanism would remain more or less constant for a given flow regime, which is consistent with field observations (Fig. 1B) but cannot be explained by the classic Liesegang banding theories^9^. For the first time, we demonstrate that Ostwald ripening in an advective flow field is able to generate chemical waves along the flow pathway. Since they are induced by water advection, such bandings can develop pervasively in a large portion of geologic formations, as observed^1^,^2^. In contrast, the classic diffusion-based Liesegang banding is generally limited in space. Because of pore confinement (Fig. 2), iron oxide precipitates in fine-grained sandstone may be subjected to continual Ostwald ripening as flow regimes change, leading to a complex set and/or sequence of pattern formation (Fig. 1A and D).

## Discussion

Using representative parameter value ranges constrained above, the typical time scale for iron oxide pattern formation (*T*_*t*_) can be estimated by 
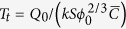
 (see Methods). It is estimated to range from tens of years to a few thousands of years, a perhaps surprising short geologic time, yet in good agreement with observations. For example, at Lake Brown in Western Australia, the diagenetic nodules were found to form within <3,000 years, based on dating of stratigraphically higher and lower beds^36^. Such a short time scale, combined with the dynamic nature of confined Ostwald ripening (Fig. 2), means that iron oxide cements and their patterns may be continually reworked over time. This evolutionary nature of iron oxide cementation is consistent with paleomagnetic measurements in Jurassic Navajo Sandstone which indicate that the Navajo has only recently acquired a new, stable secondary magnetic moment^45^. A (U-Th)/He geochronological study of iron oxide cements in sandstone of Colorado Plateau indicates that young waters (ca. 2-3 m.y.) passed through the system^46^. Similarly, the remnant magnetization in several Triassic and earliest Jurassic sediment units from the Colorado Plateau was found to be acquired over an extended time period (~35 m.y.) and not coeval with the stratigraphic age of the units^47^. The proposed concept also sheds light on a recently discovered dilemma between the latitudinal position of supercontinent Pangea reconstructed from paleomagnetic measurements and that deduced from wind directions recorded in dunes in eolian sandstones in the southwestern United States^48^. The atmospheric circulation constructed from the sand dunes shows a broad sweep of northeasterly winds over the northernmost part of the region and then curving to become northwesterly in the south part, indicating that the Jurassic sandstones were deposited close to the equator. This observation is at odds with the paleomagnetic measurements, which indicate that those sandstones were formed around the latitude of 20 ^o^N (ref. 37). Given the dynamics of continual iron oxide dissolution-precipitation in cementation as discussed above, this inconsistency can possibly be attributed to multiple diagenetic episodes with more recent ones overprinting earlier ones on rock magnetization.

The model presented above assumes an initial condition of sand grains coated with iron oxide films. Such films form by oxidation and precipitation of ferrous iron released by early alteration of detrital grains of biotite and pyroxene and/or by precipitation of dissolved Fe^3+^ species carried in by acidic (and perhaps sulfate-rich) fluids. Iron oxide patterns may immediately start to form behind either an oxidation or a precipitation front^13^. Patterning suggested above may begin with repetitive banding and then switch to nodule formation as the fluid advection abates. The same physical domain may experience multiple hydrologic regimes, leading to complex pattern formations (Fig. 1)^34^,^49^. Early formed nodules may be subjected to further differentiation inside, resulting in internal concentric banding. This differentiation can be induced by a classic counter-diffusion Ostwald ripening process. Thus, internal concentric banding may indicate the maturity of nodule evolution, as it does also in the case of mature pisolites developed in lateritic weathering profiles^50^. Note that the linear analysis presented here in general applies only to an internal region of an aquifer, which is far from a water recharge boundary so that any boundary influence becomes negligible. At the boundary, an iron oxide dissolution front may be developed, and reactive infiltration instability may then be induced^51^. The proposed confined Ostwald ripening mechanism is expected to reinforce the infiltration instability, because it tends to induce more mineral precipitation in a lower porosity (iron oxide rich) domain and more mineral dissolution in a higher porosity domain. This coupling might be responsible for the formation of scalloped (Fig. 1D) or fingered precipitates (fig. 2 in ref. 51) and pipe-like concretions^52^, and in this case concretions would tend to orient along the flow direction.

As mentioned earlier, iron oxide concretions found in Navajo Sandstone have been used as a terrestrial analogue to hematite nodules detected on Mars. The work presented in this paper may provide additional insight into extraterrestrial biosignature detection and data interpretation for Mars explorations. Small (<0.5 cm) spherical hematite nodules were found in the Eagle Crater at Meridiani Planum on Mars^3^,^53^. Sediments at this site are composed of fined-grained clastic materials derived from weathering of basaltic rocks and contain high percentages of sulfate minerals, notably jarosite [KFe^3+^_3_(OH)_6_(SO_4_)_2_] (ref. 53). Jarosite is thought to form under oxidizing acidic environments and persist under arid conditions^54^. According to our model, such chemical environments would be conducive to Fe^3+^ mobilization and therefore iron oxide pattern formation. The occurrence of nodules may indicate a stagnant hydrologic environment involved in iron oxide diagenesis (Fig. 4)^55^. New NASA Mars Science Laboratory explorations of Curiosity rover in Gale Crater indicate more diagenetic spherules and nodules^56^. Since a persistent water circulation is generally required for a sustainable subsurface life, a site with iron oxide bands, if one were found, may offer a better chance for detecting biosignatures on Mars.

Finally, many repetitive cementation patterns of various scales and mineral compositions in sedimentary deposits may also have self-organizational origins. However, each case likely has its own unique underlying mechanisms, which can only be identified by careful field and laboratory observations and by testing model predictions against observations^57^. In this sense, we caution any attempt to grossly apply the mechanism proposed in this paper to the cementation pattern formation of other mineralogies and other geological environments. For example, elongate carbonate concretions occur in fluvial sediments and their orientation has been used as a proxy to the flow field of paleoaquifers^58^. Such carbonate concretions might have formed from localized microbial reactions^59^ coupled with infiltration instability. Despite their appearance resemblance to pipe-like iron oxide nodules as discussed above, elongate carbonate nodules are likely to form from a different mechanism. It should also be recognized that the mechanism proposed here doesn’t preclude the banding formation by a classic counter-diffusion mechanism, which may occur in some circumstances where waters are stagnant, isolated redox domains may exist, and counter-diffusion of oxidant (O_2_) and reductant (Fe^2+^) could be induced. However, such bandings would generally be localized in space (not pervasive as in advection-induced bandings) and concentric in geometry with no systematic spatial orientations. Given general oxidizing conditions in red-bed formations^12^, the occurrence of these bandings in such environments is expected to be limited. We believe that pervasive iron oxide diagenetic bandings in eolian sandstone described here were predominantly formed by the proposed advection-dominated confined Ostwald ripening mechanism. Our model combines both field observations and theoretical modeling to show the complexities of the interplay among pore geometries, fluid chemistry, and flow fields in mineral dissolution and precipitation at multiple scales.

## Methods

### Scaling and asymptotic analysis

For the system of interest, the relevant time and length scales emerge from the internal dynamics of the system, and they can be determined by scaling variables in Equations (4), (5), (6), (7), (8), (9). This scaling process also allows us to lump model parameters into minimum set of dimensionless parameters. Let’s first choose a typical dissolved iron concentration (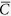) to be the solubility of iron oxide for *Q = Q*_*0*_: 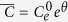. We then choose a time scale (*T*_*t*_) from Equations (5) and (8) such that a significant iron oxide dissolution and precipitation can occur over the chosen time scale: 
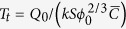
. The emerging length scale (*L*) and the typical hydraulic head (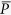) can be obtained as: 
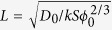
 and 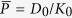, respectively, by making both the diffusion term and the advective term in equation (6) vary comparably with the chemical reaction term so that they can interact with each other, since field evidence indicates that the formation of iron oxide patterns is collectively controlled by diffusion, fluid flow and chemical reactions^1^,^2^. Accordingly, the other variables and the related mathematical operators can be scaled as follows: 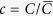, 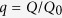, *τ *= *t*/*T*_*t*_, *x* = *X*/*L*, *u* = *VT*_*t*_*/L*, y=Y/L, 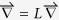, 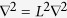, and 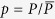. Equations (4, 5, 6, 7, 8, 9) can then be cast into a set of dimensionless scaled equations:


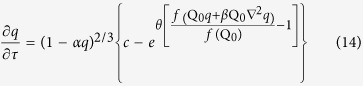



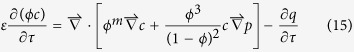



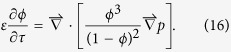



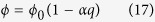


where 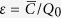. Given a typical value of 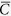 (~5 × 10^−10^ mol/cm^3^ solution, ref.^39^) and *Q*_0_ (~10^−3^ mol/cm^3^ sediment^23^), *ε* < 10^−6^, indicating that, because of the natural difference between aqueous solutions and crystalline matter, aqueous concentrations of ions (here, iron) are always orders of magnitude smaller than their concentration in minerals, i.e., the density. Therefore, the left-hand side terms in Equations (15) and (16) are negligible, and the two equations can be treated to be pseudo-steady state on the time scale of pattern formation^33^.

## Linear stability analysis

Let *ε* → 0 in equations (14) through (17). A uniform steady state solution to the equations can be found: *q*_s_ = 1, *c*_s_ = 1, *φ*_s_ = *φ*_*0*_(1–α), 
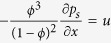
, and 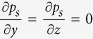. Linearizing the equations around the steady state solution with respect to perturbations *δq*, *δ*c, *δp*, and *δφ*, we obtain:


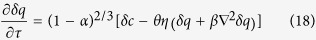



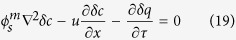



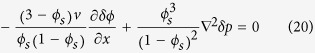



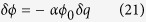


Assuming that the perturbations have the form:


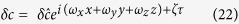



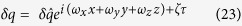



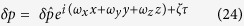



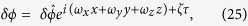


we obtain the following homogeneous algebraic equations:


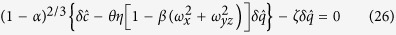



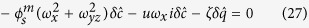



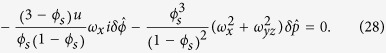



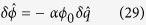


The condition for a nontrivial solution of 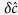, 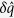, 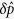 and 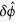 is:


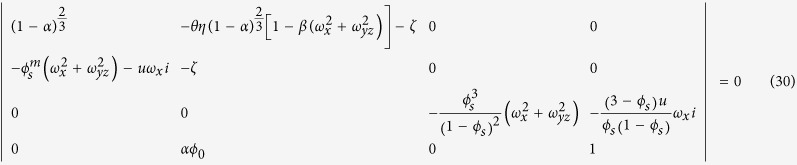


Solving the equation for *ζ*, we obtain the dispersion equations (10) and (11).

### Estimation of pattern scale

For a no-flow case (*u* = 0), from equation (10), we have


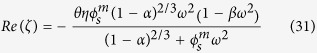


where 
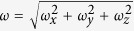
. The preferred wave number *ω*_*max*_ can be found by taking 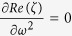:


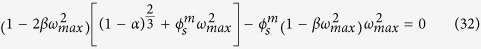


We then have:


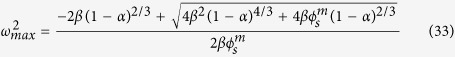


Since 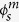 << (1 – *α*) ≈ 1 and *β* is on the order of unity, we obtain:


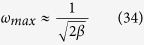


or


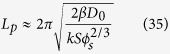


Adding the term *φ*_*s*_ in the nominator to convert the unit of cm^3^ bulk sediment to the unit of cm^3^ solution, we obtain Equation (12).

For a flow case with a large *u*, since *ω*_*max,*2_ = 0 (Fig. 3), from equation (10), we have


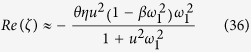


Similarly, the preferred wave number *ω*_*max*,1_ can be found by taking 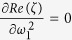:


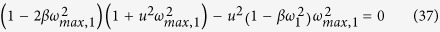


Solving for 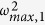, we obtain:


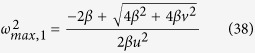


We then have:


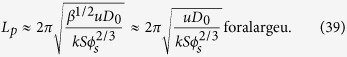


Adding the factor *φ*_*s*_ to convert the unit of cm^3^ bulk sediment to the unit of cm^3^ solution, we obtain Equation (13).

## Symbols

*B* – Constant characterizing the influence of mineral distribution mineral solubility

*C* – Dissolved iron concentration in pore water

*C*_*e*_ – Solubility of iron oxide particles


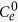
 – Solubility of the bulk phase


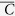
 – Typical dissolved iron concentration

*c* – Scaled dissolved iron concentration

*c*_*s*_ – Scaled dissolved iron concentration at a steady state

*D* – Effective diffusion coefficient

*D*_0_ – Diffusion coefficient of dissolved iron species

*f* – Functional dependence of Gibbs free energy on weighted iron oxide content

*K* – Hydraulic conductivity

*k* – Reaction rate constant

*K*_*r*_ – Kernel function describing the textural dependence of Gibbs free energy of iron oxide

*L* – Typical length

*L*_*p*_ – Wave length of cementation pattern

*P* – Hydraulic head


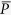
 – Typical hydraulic head

*p* – Scaled hydraulic head

*p*_s_ – Scaled hydraulic head at a steady state

*Q* – Local iron oxide content in bulk rock


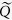
 – Weighted iron oxide content within the neighborhood

*Q*_*0*_ – Initial iron oxide content

*q* – Scaled iron oxide content

*q*_s_ – Scaled iron oxide content at a steady state

*R* – Gas constant

*R*_*p*_ – Precipitation rate of iron oxide

*r*_*p*_ – Radius of iron oxide particles


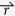
 – Spatial coordinate

*S* – Geometric factor of pores

*T* – Absolute temperature

*T*_*t*_ – Typical time

*V* – Groundwater velocity

*V*_*m*_ – Molar volume of the solid

*u* – Scaled groundwater velocity

*u*_*c*_ – Scaled critical groundwater velocity for pattern transition

X, Y, Z – Spatial coordinate

x, y, z – Scaled spatial coordinate

*α* − *Q*_0_*V*_*m*_/*φ*_*0*_

*β* − *B*/*L*^2^


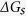
 – Excess Gibbs free energy of iron oxide film

*δq*, *δc*, *δp*, *δφ* – Perturbations of *q*, *c*, *p*, and *φ* respectively


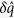
, 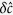, 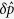, 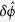 – Perturbation amplitudes of *q*, *c*, *p*, and *φ* respectively


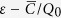


*ζ* – Growth rate of a perturbation

*η *− *f*′(*Q*_0_)*Q*_0_/*f*(*Q*_0_)

*θ* − *f*(*Q*_0_)/*RT*

σ – Surface energy between iron oxide and water

*τ* – Scaled time

*ω*_*x*_, *ω*_*y*_, *ω*_*z*_ – Wave number vector of a perturbation

*ω*_*max,x*_, *ω*_*max,y*_, *ω*_*max,z*_ – Wave number vector corresponding to the maximum perturbation growth rate

*φ* – Porosity

*φ*_*0*_ – Porosity of sandstone without iron oxide cementation

*φ*_*s*_ − *φ*_0_(1 − *α*)

## Additional Information

**How to cite this article**: Wang, Y. *et al.* Self-organized iron-oxide cementation geometry as an indicator of paleo-flows. *Sci. Rep.* **5**, 10792; doi: 10.1038/srep10792 (2015).

## Supplementary Material

Supplementary Information
