# Supplementary material for: Self-organized iron-oxide cementation geometry as an indicator of paleo-flows
Source: Sci Rep. 2015 Jun 30;5:10792. doi: 10.1038/srep10792 (PMC4485175; doi:10.1038/srep10792)
Supplement: Supplementary Information [file srep10792-s1.pdf]

# Supplementary Information

## Self-organized iron-oxide cementation geometry as indicators of paleo-flows

Yifeng Wang[1*], Marjorie A. Chan[2] & Enrique Merino[3]

[1]Sandia National Laboratories, P. O. Box 5800, Albuquerque, New Mexico 87185-0779, USA
[2]Department of Geology and Geophysics, University Utah, Salt Lake City, Utah 84112-0111, USA
[3]Department of Geological Sciences, Indiana University, Bloomington, Indiana 47405, USA

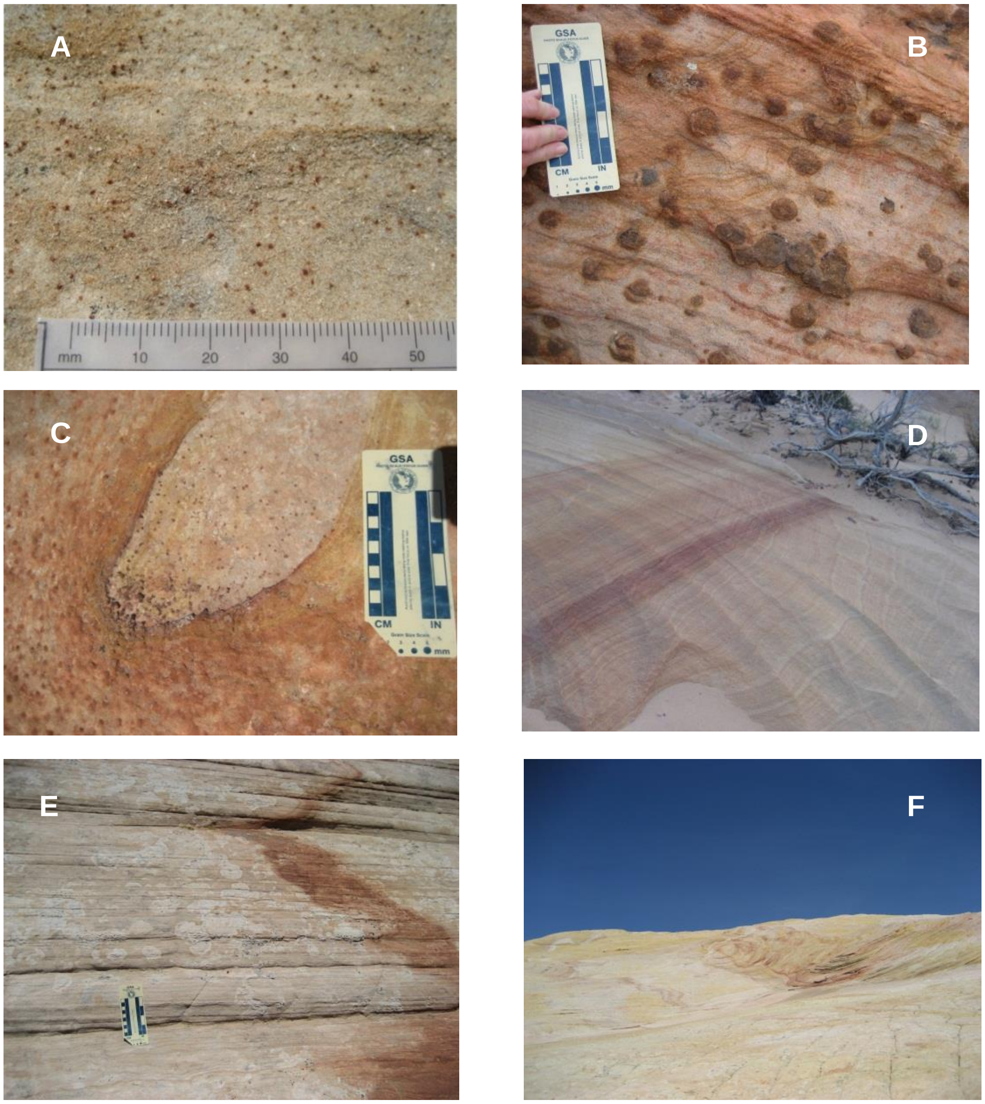


**Figure S1 | Additional images of iron oxide pattern formation in fine-grained Jurassic Navajo Sandstone.** (A) Very small and abundant mm-sized "micro-concretions" cemented by iron oxides. Locality: Cottonwood Wash area, Grand Staircase Escalante National Monument, Utah. (B) Centimeter-sized iron oxide concretions in a chemical reaction front localized towards the base of an eolian dune set. Locality: Snow Canyon State Park, Utah. (C) Small iron oxide concretions and Liesegang banding. Locality: Coyote Buttes area, Vermilion Cliffs National Monument, Utah. (D) Sub-meter scale banding

with finer scale internal nested Liesegang banding, where field of view ~ 2 m across.  Locality: Paria Plateau area, Vermilion Cliffs National Monument, Arizona.  (E) Sub-meter scale  iron oxide banding. Locality: Cottonwood Wash area, Grand Staircase Escalante National Monument, Utah.  (F)  Large, meter-scale diffuse iron oxide banding patterns in bleached sandstone. Locality: Cottonwood Wash area, Grand Staircase Escalante National Monument, Utah.
